# Using species distribution models to define nesting habitat of the eastern metapopulation of double‐crested cormorants

**DOI:** 10.1002/ece3.2620

**Published:** 2016-12-20

**Authors:** Kate L. Sheehan, Samuel T. Esswein, Brian S. Dorr, Greg K. Yarrow, Ron J. Johnson

**Affiliations:** ^1^ Scripps Institution of OceanographyUniversity of California San DiegoLa JollaCAUSA; ^2^ Department of Forestry and Environmental ConservationClemson UniversityClemsonSCUSA; ^3^ U.S. Department of AgricultureAnimal Plant Health Inspection ServiceWildlife ServicesNational Wildlife Research CenterMississippi StateMSUSA

**Keywords:** conservation, cormorant, metapopulation, nesting habitat, species distribution model, wildlife management

## Abstract

When organisms with similar phenotypes have conflicting management and conservation initiatives, approaches are needed to differentiate among subpopulations or discrete groups. For example, the eastern metapopulation of the double‐crested cormorant (*Phalacrocorax auritus*) has a migratory phenotype that is culled because they are viewed as a threat to commercial and natural resources, whereas resident birds are targeted for conservation. Understanding the distinct breeding habitats of resident versus migratory cormorants would aid in identification and management decisions. Here, we use species distribution models (SDM: Maxent) of cormorant nesting habitat to examine the eastern *P. auritus* metapopulation and the predicted breeding sites of its phenotypes. We then estimate the phenotypic identity of breeding colonies of cormorants where management plans are being developed. We transferred SDMs trained on data from resident bird colonies in Florida and migratory bird colonies in Minnesota to South Carolina in an effort to identify the phenotype of breeding cormorants there based on the local landscape characteristics. Nesting habitat characteristics of cormorant colonies in South Carolina more closely resembled those of the Florida phenotype than those of birds of the Minnesota phenotype. The presence of the resident phenotype in summer suggests that migratory and resident cormorants will co‐occur in South Carolina in winter. Thus, there is an opportunity for separate management strategies for the two phenotypes in that state. We found differences in nesting habitat characteristics that could be used to refine management strategies and reduce human conflicts with abundant winter migrants and, at the same time, conserve less common colonies of resident cormorants. The models we use here show potential for advancing the study of geographically overlapping phenotypes with differing conservation and management priorities.

## 1 Introduction

Wildlife management initiatives are often developed to protect individual species, subspecies, or populations (Blair, Gutierrez‐Espeleta, & Melnick, 2013; Groom, Meffe, & Carroll, 2006). At times, however, subpopulations (local populations of a metapopulation; Hanski & Gilpin, 1991) or discrete groups within a species (phenotypes) may be difficult to differentiate and have conflicting management or conservation goals. Differentiation of subgroups with similar phenotypes, however, might be possible using their seasonal distributions and behavioral traits such as foraging patterns and preferences. For example, thousands of migratory double‐crested cormorants *Phalacrocorax auritus* that winter on lakes in South Carolina are viewed as a threat to commercial and natural resources, whereas small colonies of cormorants that breed in the state during the summer are viewed as favorable contributors to ecosystem processes and need conservation. Resource managers in South Carolina were tasked to develop management strategies to limit the negative impacts of the migratory cormorants and, at the same time, to conserve the year‐round colonies (personal communication, D. Shipes, SCDNR). Understanding the distinct breeding patterns and habitats of resident versus migratory cormorants could help identify the subgroups and inform the conservation and management plans (Carranza & Winn, 1954; Fonteneau, Paillisson, & Marion, 2009).

Human–wildlife conflicts over shared fisheries resources have precipitated dozens of culling programs for *P. auritus*, killing thousands of birds annually to reduce competition for sport and forage fishes (Dorr, Hatch, & Weseloh, 2014). Once distributed ubiquitously throughout North America, cormorant nesting colonies were documented along nearly all freshwater and coastal habitats (Audubon, 1840–1844). Cormorant population bottlenecks occurred in the twentieth century when their abundance in North America declined from millions to thousands (Dorr et al., 2014; Wires & Cuthbert, 2006). During this population decline, two phenotypes (suggested by some to be distinct subspecies) of the eastern population became apparent: a migratory group that breeds in the northern United States and Canada and a resident group that breeds in the south. Both groups winter in the southeastern United States and Mexico. During their population bottlenecks, there were no breeding cormorants in the state of South Carolina. Although *P. auritus* populations are now considered to be recovered (Dorr et al., 2014), it is unclear whether contemporary breeding colonies in South Carolina can justifiably be managed separately from migratory birds.

A group of cormorants sometimes referred to as the Florida subspecies (Forrester et al., 2003; Hatch, 1995; Post, 1988) is thought to be re‐expanding northward, and managers suggest that birds in South Carolina may belong to this group (F.J. Cuthbert et al. 2011—unpublished data MNDNR). Others, however, have suggested the migratory phenotype is cueing in on the large lake systems (like habitats found in northern breeding sites) and reducing their migration distances (Post & Post, 1988; Post & Seals, 1991). The debate over the status of contemporary southern breeding birds remains contentious as molecular studies have yet to successfully differentiate among breeding phenotypes of *P. auritus* in eastern North America (Green, Waits, Avery, & Leberg, 2006; Mercer, Haig, & Roby, 2013; Waits, Avery, Tobin, & Leberg, 2003). Sheehan, Tonkyn, Yarrow, and Johnson (2016b) used intestinal parasite assemblages as evidence that resident and migratory birds forage together in Mississippi and Alabama during the winter. If this is the case in South Carolina, lethal management of cormorants in winter risks the concurrent removal of the local birds that breed in that state. Parasite community data are not yet available for birds breeding or wintering in South Carolina and require lethal means to obtain. Therefore, we sought nonlethal species distribution modeling (SDM) methods to define the migratory and resident phenotypes of cormorants to better identify and categorize the cormorant colonies nesting in South Carolina.

Without an official designation of subspecies, we consider the two groups of cormorants (northern breeding and southern breeding) to be phenotypes occurring within a metapopulation. The use of the metapopulation concept is fitting for *P. auritus* because they live in fragmented landscapes, their suitable habitat (water) is limited and occurs in discrete fragments (Hanski, Mononen, & Ovaskainen, 2011; Ojanen et al. 2013), and their population dynamics are considered to be independent (Hanski, 2004). We have found no evidence that birds commonly switch between northern and southern breeding, although banding evidence suggests imperfect fidelity among these groups (personal observation, B. Dorr, USDA/APHIS/NWRC). Thus, we consider migration behavior a phenotypic characteristic that differentiates the two groups (Hanski, 2004).

Migration behavior is used to define groups of birds for conservation and management. In Mississippi, resident Mississippi sandhill cranes (*Grus canadensis pulla*) are listed as endangered (Henkel et al., 2012) and consequently are conserved, while migratory birds elsewhere are hunted for sport (Raftovich, Chandler, & Wilkins, 2015). Similarly, some migratory Canada Geese are protected from hunting and harassment, while culling programs of resident birds are commonplace (Beston, Williams, Nichols, & Castelli, 2015; Holevinski, Malecki, & Curtis, 2006; Nichols, 2014). Because genetic distinction within the aforementioned metapopulations is questionable or nonexistent (Glenn, Thompson, Ballard, Roberson, & French, 2002; Smith, Craven, & Curtis, 1999), distinguishing phenotypes based on the behavior is an accepted form of differentiation for conservation and management planning. Migration and breeding behaviors are influenced by climatic, geologic, biologic, and anthropogenic factors (Guillaumet et al., 2011; Hutto, 1985; Walther et al., 2002), and if truly different, we expect migratory and resident groups of *P. auritus* to respond to these variables in distinct ways. Using the environmental characteristics of known nesting sites of cormorants, we developed two ecological niche models to describe the habitat of resident and migratory *P. auritus* during the breeding season. If the two phenotypes are to be managed differently throughout their range, the methods used here could be considered in other situations where the identity of breeding cormorants is in question.

Species distribution models can be used to predict future, current, and past distributions of species, such as current distributions of rare or cryptic species (Engler, Guisan, & Rechsteiner, 2004), potential distributions of invasive species (Young, Abbott, Caldwell, & Schrader, 2013), and future distributions of organisms in relation to climate change (Thomas et al., 2004). The types of data input into SDM can limit the statistical assessments used to develop predictive models (Aarts, Fieberg, & Matthiopoulos, 2012; Hastie & Fithian, 2013). Using presence–absence and abundance data with true records of absences are ideal for SDM development (Howard, Stephens, Pearce‐Higgins, Gregory, & Willis, 2014; Van Couwenberghe, Collet, Plerrat, Verheyen, & Gegout, 2013), but the availability of such data is limited. Nonetheless, presence‐only datasets can still be used in a presence–absence assessment by assuming all locations not listed as presence points are absence points (Phillips & Dudik, 2008). This presence‐inferred‐absence method requires reliable presence data to ensure that true presence points are not included in the absence dataset (Guisan & Thuiller, 2005) and to identify what conditions exist at occurrence sites that do not occur elsewhere in the landscape. Maximum entropy (Maxent) is an increasingly popular method for developing predictive SDMs based on the presence‐only data (Evans et al., 2010; Phillips & Dudik, 2008), and its applicability and methodology are well documented (Oppel et al., 2012; Peterson, Papes, & Eaton, 2007; Renner & Warton, 2013).

In this study, we use SDM predictions, to better identify which phenotype(s) are likely to be breeding in a state where conservation and management priorities require reliable predictions. We developed SDMs from environmental variables expected to be important for successful breeding of resident and migratory *P. auritus* subpopulations. Breeding waterbird colonies are relatively conspicuous, and there is a low likelihood of missed detection (Ridgway, 2010); thus, presence‐inferred‐absence models are suitable for modeling *P. auritus* nesting habitat distributions. We hypothesized that the habitat of known breeding sites for migratory and resident phenotypes would be significantly different from the general landscapes within Minnesota and Florida, respectively, and that landscape variables important for the prediction of nesting sites would be associated with waterways, fisheries, and avian mortality. We confirmed model predictions using contemporary breeding colony data of *P. auritus* within the states of Minnesota and Florida using observed presence, absence, and colony size data. We hypothesized that the prediction values provided by the models would correlate with the size of a cormorant colony. Lastly, we use the models trained on Minnesota and Florida nesting data to predict contemporary nesting sites for resident and migratory cormorants in South Carolina.

## 2 Methods

### 2.1 Nesting colony data

We developed nesting habitat models for migratory *P. auritus* using available nesting survey data from Minnesota (1977–2010: Guillaumet, Dorr, Wang, & Doyle, 2013; Wires & Cuthbert, 2006; Dorr et al., 2014) and resident *P. auritus* in Florida (1970–1999: Nisbet et al., 2002). These states are historical breeding areas for migratory and resident cormorant phenotypes, respectively. Data for nesting sites in South Carolina were based on the colonies documented by SCDNR in 2011 and 2012 (unpublished data, breeding bird database accessed October 2013, SCDNR), publications reporting contemporary nesting locations (Post & Seals, 1991), and personal observations from the field (unpublished data, K. Sheehan, Clemson University, 2011–2013). All count data were converted to presence points for Maxent model creation and presence/absence for model validation.

**Figure 1:**
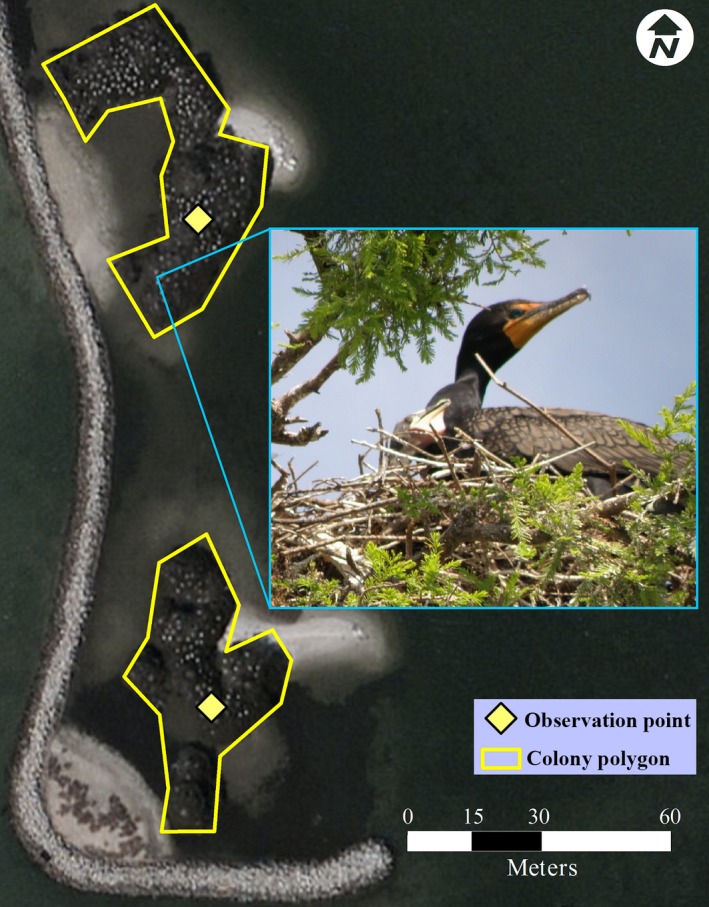
Conversion of nesting points to polygons. Aerial imagery of island nesting sites reported in Florida where sandy and shell hash substrates (light areas in the photograph) connect *P. auritus* nesting areas (inset image) in trees. Example polygons drawn around *P. auritus* colonies. Albers Equal Area Conic projection

Each colony location was initially reported as a single point despite multiple habitat characteristics occurring within a nesting site. For example, at a 30‐m resolution, a single colony could occur in forested, undeveloped, and wetland habitat. To capture the full range of environmental characteristics within each colony, we converted point data to polygons, using automated and manual methods. This also allowed us to overcome geographic positioning errors (Naimi, Skidmore, Groen, & Hamm, 2011) that associated some colonies with unlikely nesting habitats (e.g., open water adjacent to island nesting sites). Geospatial analysis of water layers from the National Hydrography Dataset (NHD) was used to identify areas of land that were <10,000 km^2^ and surrounded by water. The resulting polygons were spatially joined with nesting colony data. Manual validations were performed by overlaying colony polygons on satellite imagery; we manually adjusted the colony extent to accommodate sites not captured during automation, as was the case when rookeries occupied islands smaller than the spatial resolution of the source dataset (30 m × 30 m; Figure 1) or where rookeries occurred in swamps or mainland peninsulas. The polygon layer containing colony data was converted into a raster with each 30 m × 30 m cell representing the presence or absence of a nesting colony. And finally, each presence cell was converted to point data (one point created at the center of each 30 m × 30 m cell) for input into the Maxent analysis. Because most colonies were represented by multiple points, potential spatial autocorrelation could inflate the fit of our models (Hijmans, 2012). We tested model residuals for spatial autocorrelation using a 100‐permutation Mantel test on distance matrices of geographic covariance and residual covariance. To overcome issues of spatial autocorrelation, we validated our models in three ways: (1) We verified low prediction probability of observed absence points (created in the same way as present points); (2) we created random landscape models to verify that the habitat used by *P. auritus* was distinct from habitat throughout the rest of the state; and (3) we transferred the models (based on the observed presence, observed absence, and random data points) between all states to verify that the predicted habitat models were unique to each specified circumstance.

### 2.2 Layer development for individual parameters

**Figure 2:**
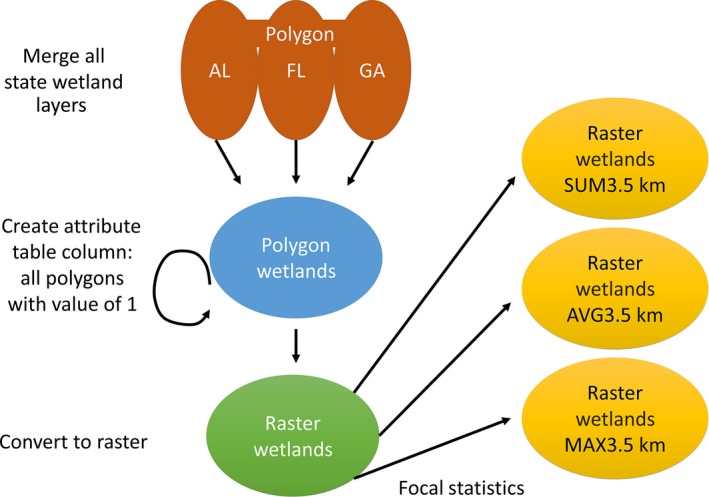
Derivation steps for wetland‐related layers used to develop the Maxent model trained on resident cormorants in Florida. Although their base layers used different in geographic extents, the same methods were used to develop wetland‐related layers for the states of Minnesota and South Carolina

The selection of nesting locations by *P. auritus* could be associated with foraging habitat, nesting habitat, and/or anthropocentric parameters. These variables were derived from data layers obtained through publicly available Web downloads from the National Atlas, National Land Cover Database (NLCD), National Wetlands Inventory, and the NHD (see Table S1 in Appendix S1 in Supporting Information). Fish consumption advisories and avian disease outbreaks were obtained from the Environmental Protection Agency, and fish stocking activity data were obtained from each state's fisheries agency. We obtained climate data from the PRISM Climate Group, Oregon State University. Because downloaded data were in different formats (raster, polygon, point, polyline) and sometimes were split into multiple files, each parameter was individually processed to obtain similarity in transformation, registry, and raster cell value. In some cases, this required simply snapping raster data to a common registry point (e.g., climate data), and in other cases, spatial joining of data and object classes (e.g., wetland data), raster conversion (e.g., fish advisories), and raster reclassification (e.g., land use data) was needed to achieve consistent and comparable formatting (parameter processing details appear in Appendix S1). We provide a schematic example of layer development for wetland data layers (Figure 2).

### 2.3 Derivation of parameters

Guisan and Thuiller (2005) recommend focal statistics for highly mobile organisms because observations are likely to vary between potential and realized distributions. The likelihood of any given focal cell to be impacted positively or negatively by the values of other nearby cells was either summed, averaged, or maximized at a radius of either 3.5 or 10 km. We based focal radii on foraging distances reported during the breeding season (Coleman, Richmond, Rudstam, & Mattison, 2005; Dorr et al., 2012; Sheehan, Hanson‐Dorr, Dorr, Yarrow & Johnson, 2016a). The final state‐based raster layers were converted to tagged image file format (tiff) and imported into the R statistical computing environment (r‐project.org) with the Maxent Java application (cs.princeton.edu/~schapire/maxent/) using the “dismo” package (Hijmans & Elith, 2013). To capture biological processes that occur at differing spatial extents, multiple focal statistics were calculated for many environmental variables. As such, we expected these layers to covary and identified groups of correlated parameters during model development (derived from the same initial dataset or source agency; see Table S2 in Appendix S1). Within each group, the variable that explained the most variance in nesting site distribution was retained and the remaining group members were removed from the model (York et al., 2011; Young et al., 2013).

### 2.4 Species distribution models

**Figure 3:**
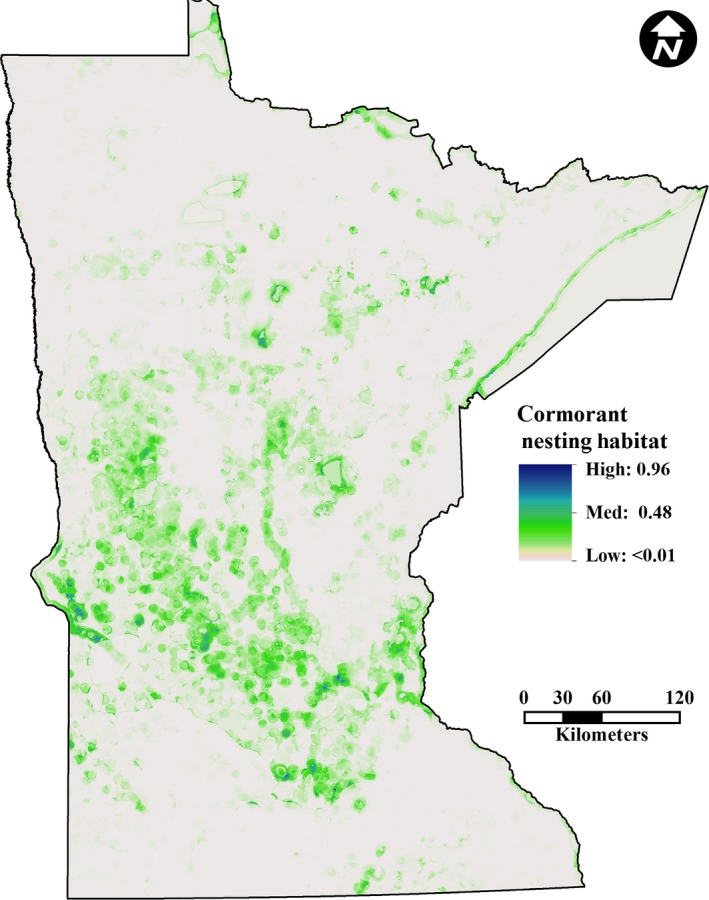
Prediction of suitable cormorant nesting habitat in Minnesota. Albers Equal Area Conic projection

**Figure 4:**
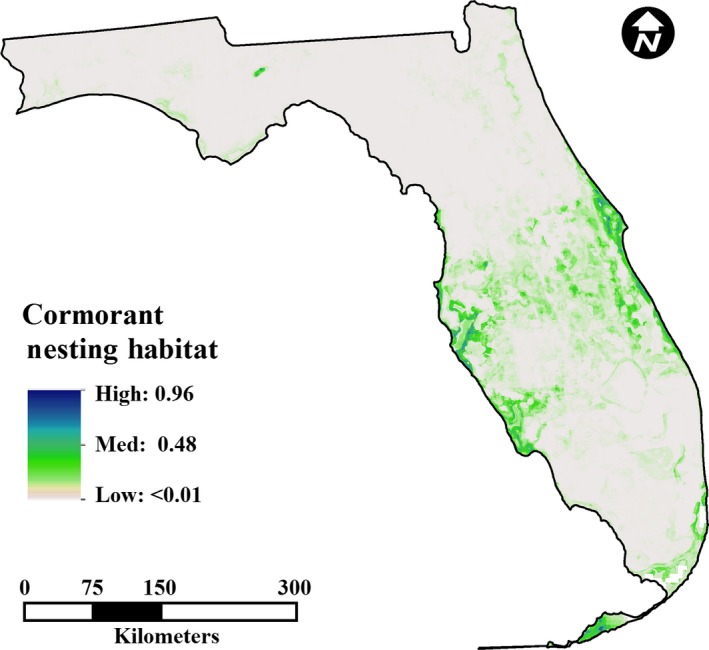
Prediction of suitable cormorant nesting habitat in Florida. Albers Equal Area Conic projection

**Figure 5:**
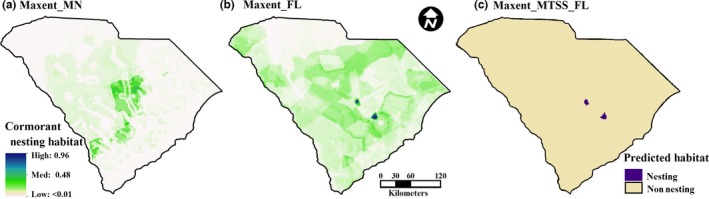
Prediction of suitable nesting habitat in South Carolina**.** Prediction values of *P. auritus* based on the parameters that describe the ecological niche of cormorants nesting in (a) Minnesota, (b) Florida, and (c) the MTSS threshold value for Florida altering continuous predicted values to suitable (good) nesting habitat and unsuitable (poor) habitat. Albers Equal Area Conic projection

We assessed the influence of environmental parameters (derived parameters) of the entire extent of each state for nest site selection of *P. auritus* in Minnesota and Florida. We stacked all derived variables (Phillips, Anderson, & Schapire, 2006) and, using default Maxent algorithms, reduced the number of model parameters using three series of five iterations, removing environmental variables in the following order: variables contributing no explanatory power to the model (providing 0% contribution); variables providing 0.5% or less explanatory contribution (Holt, Salkeld, Fritz, Tucker, & Gong, 2009); and variables that covaried significantly with parameters with greater explanatory power (Koncki and Aronson 2015; see Table S2 in Appendix S1). By removing nonexplanatory and redundant variables, we reduced model parameters and overfitting (Merckx, Steyaert, Vanreusel, Vincx, & Vanaverbeke, 2011). The models trained on Minnesota and Florida nesting data were used to generate predicted geographic distributions within the political boundaries of each state (Minnesota, Figure 3; Florida, Figure 4; and South Carolina, Figure 5).

### 2.5 Testing model predictions

In addition to common predictors of model success (receiver operating characteristic area under the curve [AUC], which is the likelihood of a model to assign a higher prediction value for any randomly chosen presence point when compared to any randomly chosen background point; Merow, Smith, & Silander, 2013), we used observed absence data points in Minnesota and Florida (Nisbet et al., 2002; Wires, Cuthbert, & Hamilton, 2011; Wires, Haws, & Cuthbert, 2005) to test the predictive ability of each model. We sampled point data from the predictive map outputs based on the observed presence and absence points for each state and compared prediction values with empirical presence–absence data. Thresholds of the maximum training sensitivity plus specificity (MTSS) values (Jimenez‐Valverde & Lobo, 2007) were used to transform continuous data to binary, where each point was categorized as nesting habitat (1) or non‐nesting habitat (0) of *P. auritus* (Cao et al., 2013). Chi‐square analyses of actual versus predicted data were used to assess the ability of each model to correctly predict the status of a site observed for nesting *P. auritus*. We also assessed whether model output was a good predictor of colony size and used linear regressions comparing nest density (nests/km^2^) to prediction values derived by the Minnesota and Florida models.

To confirm that predictor variables in nesting models were not merely a representation of the landscape, we created a random set of 5,000 points within the state boundaries of Minnesota and Florida and developed null models in the same manner as the empirical models created with the presence data. These predictive maps of the random landscape developed from the null models were compared to empirical nesting census data for Minnesota, Florida, and South Carolina. The null models aided in confirming that our models trained on the observed presence data were accurate despite potential spatial autocorrelation (Hijmans, 2012).

## 3 Results

### 3.1 Statewide models

The Maxent model for migratory cormorant nesting habitat in Minnesota produced prediction values ranging from <0.010 to 0.956 (Figure 3). The AUC value was 0.911, indicating the excellent model fit; however, as expected, residuals exhibited spatial autocorrelation (Mantel test, *p* = 0.001). The *t* tests performed on the observed field data confirmed the prediction success of this model for migratory birds (*p *< 0.0001, Table 1) with a mean prediction of absence sites of 0.12 (95% CI: 0.108–0.133) and presence sites of 0.54 (95% CI: 0.535–0.544). The threshold for the MTSS as determined by Maxent algorithm was 0.277 (Chi‐square *p* < 0.0001, *R*‐square = 0.814). The Maxent predictor values were not a strong estimate of colony size, as higher prediction values (those most likely to be *P. auritus* nesting sites) corresponded with small colony sizes rather than large colony sizes (*p* = 0.0278, *R*‐square = 0.002).

**Table 1:** Results of Student's *t* tests comparing model output based on the prediction value and values truncated at the threshold for maximum training sensitivity plus specificity (MTSS)

Model	State test	*p*‐value	*R*‐square	Threshold	
				*p*‐value	*R*‐square
Minnesota	Minnesota	<0.0001	0.653	<0.0001	0.750
Florida	Florida	<0.0001	0.791	<0.0001	0.827
Minnesota	Florida	<0.0001	0.082	N/A	N/A
Florida	Minnesota	0.507	0.0004	<0.0001	0.004
Minnesota	S. Carolina	<0.0001	0.256	N/A	N/A
Florida	S. Carolina	<0.0001	0.218	<0.0001	0.036

The degree of freedom for all tests was 1.

The Florida model (Figure 4) successfully predicted the presence and absence of *P. auritus* nesting colonies (*p* < 0.0001, Table 1, AUC of 0.887, Mantel test for autocorrelation *p* = 0.001). The mean prediction value for absence sites was 0.040 (95% CI: 0.0360–0.0450) and for presence sites was 0.496 (95% CI: 0.491–0.500). The MTSS threshold value used for presence/absence designation of a site was 0.298 (Chi‐square *p* < 0.0001, *R*‐square = 0.827). A positive regression of Florida colony densities agreed with prediction values derived from Maxent (*p* < 0.0001, *R*‐square = 0.203).

Seventeen parameters were included in the final models predicting *P. auritus* nesting habitat in Minnesota and Florida (Table 2). Of these, eight variables occurred in both models including variables important for cormorant foraging, nesting, and anthropogenic factors.

**Table 2:** Variable contribution for parameters included in the final cormorant nesting habitat models developed with Maxent in Minnesota and Florida

		Minnesota	Florida
Foraging	Avg. Wetland Area	4^a^	6.9^a^
	Lbs. Fish Stocked 10k		
	Lbs. Fish Stocked 3.5k	0.7	
	Min Temp September		
	Num Fish Stocked 10k		
	Water Availability 3.5k	23.3	
	Water Presence 10k		
	Water Quantity 3.5k	16.5	
	Water Quantity 10k		19.7
Nesting	Forested Land	9.3^a^	11^a^
	Max Temp June	1.8^a^	2.6^a^
	Undeveloped Land	2.7^a^	16.1^a^
	Max Temp March		3.7
	Max Temp September		
	Min Temp March	1.3^a^	12^a^
	Min Temp September	4.4	
	Min Tempt June		2.9
	Precipitation March	3.4	
	Precipitation September	5.3	
	Avian Botulism Death		0.1
Anthropocentric	Anthropogenic Land		
	Agriculture Quantity		12
	Avian Lead Poisoning		
	Avian Pesticide Death	1.1^a^	0.7^a^
	Human Pop. Density		2.1
	Impervious Surf. Quant	13.4^a^	4.8^a^
	Indian Land	1	
	Land Use Change	1.9^a^	2.7^a^
	Mercury Fish Advisory	7.3^a^	0.6^a^
	Rescinded Fish Adv.	2.7^a^	2^a^

a Factors that appear in both models.

### 3.2 Model predictions for South Carolina

Contemporary colonies of *P. auritus* nesting in South Carolina persist in and around reservoir lakes created in the 1950s (Post & Post, 1988; Post & Seals, 1991; personal observation, K. Sheehan, Clemson University). The models for Minnesota (Figure 5a) and Florida (Figure 5b) performed well when transferred to predict nesting sites in South Carolina (Table 1); however, prediction values based on the Minnesota model were low. And when truncated with threshold values, the Minnesota model yielded no predicted nesting sites. Nesting sites of *P. auritus* based on the Florida model identified two colonies with the MTSS threshold values (Figure 5c; *p* < 0.0001, *R*‐square = 0.3401) where cormorants currently breed.

### 3.3 Model validation results

The residuals of our models were spatially autocorrelated (migratory *P. auritus* Mantel r statistic = 0.103, *p *= 0.001; resident *P. auritus* Mantel *r* statistic = 0.255, *p *= 0.001). In light of these results, cross‐validation and null model tests were important to confirm model performance. When tested for prediction success in Florida, the Minnesota model was significant (*p* < 0.0001), but explained little of the variance in nest presence (R‐square = 0.082). When converted to the presence/absence of nesting habitat using the MTSS threshold, the Minnesota model identified no *P. auritus* nesting sites present in Florida. Likewise, the prediction values for Florida model did not successfully predict nesting locations in Minnesota (*p *= 0.507) and correctly identified few nesting locations with MTSS threshold values (*p *= 0.004, R‐square = 0.004). The lack of fit between the two phenotype models suggests that these groups cue in on landscape characteristics differently. To ensure that model variance did not simply correspond with overall differences in landscape characteristics, null models built from randomly generated presence points were developed. The null Minnesota model did not successfully predict nesting sites of *P. auritus* (*p *= 0.413, AUC = 0.527). The Florida model created with random points predicted nesting habitat of *P. auritus* (*p *= 0.033, R‐square = 0.001, AUC = 0.536); however, the prediction values for absence points (mean = 0.942, 95% CI = 0.932–0.951) were higher than those for presence points (0.927, 95% CI = 0.918–0.937). Using MTSS and balanced threshold values for nesting habitat in South Carolina yielded no suitable nesting habitat based on the random Minnesota and Florida null models.

## 4 Discussion

The Maxent species distribution models developed here were predictive of breeding habitat for migratory cormorants in Minnesota and resident cormorants in Florida and had good model fit. When transferred to South Carolina, the Florida model correctly predicted the presence and absence of *P. auritus* nesting colonies and indicated that nesting habitat selection characteristics for the resident phenotype are present in South Carolina (Figure 5). In contrast, the Minnesota model was not predictive of *P. auritus* nesting colonies or nesting habitat selection characteristics in South Carolina. We used the Maxent algorithm to assess landscape characteristics in Minnesota and Florida based on the suitability of local parameters important for the foraging and nesting success of resident and migratory cormorant phenotypes. We detected similar variables in the models trained on data from Minnesota and Florida, but the importance of each parameter varied. Effective management of wildlife metapopulations that have phenotypes with differing conservation imperatives requires a thorough understanding of landscape features that could promote or discourage the establishment of local populations. The inclusion of specific variables in both models highlights the importance of the landscape parameters selected in our models and could be investigated in greater detail to improve conservation and management plans for suites of wildlife species (Guisan & Thuiller, 2005; Nicholson et al., 2006) including waterbirds.

### 4.1 Considerations for other waterbird distribution models

Understanding the mechanistic biology and ecology of other nesting waterbirds will almost certainly require different local variables of importance. Specific parameters that could be informative include fine‐scale submerged and emergent vegetation data, climate conditions that would contribute to exposure severity such as lake fetch and forest cover density, and recreation variables that might be useful for estimating the anthropogenic use of each water body (water depth, boat launches, beaches, industry, etc.). *P. auritus* often nest near conspecifics and other waterbirds, and information regarding nesting sites of other bird species could be used to better inform models. We did not include conspecific data for the models presented here, because previous occurrence data collected over long periods of time were not readily accessible within all regions examined. Nonetheless, the landscape‐derived variables in our models are generally available so that the methods used here can be considered in future applications.

To expand on the methods used here, we suggest that future studies train SDMs with occupancy data from telemetered resident and migratory birds. This would allow managers to identify the habitat used during the nonbreeding season, when subpopulations overlap geographically and when management activities directly impact multiple groups or organisms. If these tracking studies were combined with SDMs, wildlife management programs could better identify critical habitats and geographic areas to target for subspecies/subpopulation conservation and management activities (Venier, Pearce, McKee, McKenney, & Nieme, 2004). Additionally, changes in migration and breeding behaviors resulting from climatic, geologic, biologic (e.g., disease), and anthropogenic changes in the landscape (Huntley et al., 2006; Kavanagh & Bamkin, 1995) could be predicted using SDMs (Peterson, 2001). Using the environmental characteristics of the present distributions of wildlife can help develop management plans to accommodate transitions through a changing landscape, effectively promoting proactive rather than reactive management (Lotter & le Maitre, 2014; May, Page, & Fleming, 2016). For these predictive models to accurately describe potential distributions, consistency among parameter variables used to train models and those to which models are transferred is critical.

When transferring models trained on data from Minnesota and Florida to the extent of South Carolina, only nesting habitat for the Florida phenotype was predicted. Transferability of a model is dependent on the similarities between the region used to develop the model and the area the model is transferred to (Peterson et al., 2007; Randin et al., 2006; Wolmarans, Robertson, & van Rensburg, 2010). In our models, data that might have differed significantly in range were ranked prior to focal statistic transformation (Table S3 in Appendix S1). This allowed for the values derived from focal statistics to be similar in range, preventing transferability problems such as interpretation errors associated with clamping (Phillips et al., 2006). Climate variables were the only environmental parameters not treated with focal statistics. Temperature averages in March and September are higher for the two southern states (South Carolina and Florida) than for Minnesota (Easterling et al., 1997); however, temperature variables in the Minnesota model contributed a cumulative 10% to the prediction value assignments. Thus, we do not expect this model to be incompatible with the variable values for South Carolina or Florida and note that springtime temperature has been identified as an important contributor to passerine species distribution models (Virkkala, Louto, Heikkinen, & Leikola, 2005). We suggest that the results of the threshold tests in South Carolina are valid and conclude that separate management and conservation goals for the nesting colonies of cormorants in South Carolina are justified.

### 4.2 Conclusions and management implications

*Phalacrocorax auritus* has a salacious history in North America where harassment and exploitation of nesting colonies by humans were historically common (Wires & Cuthbert, 2006). Cormorants are subject to various laws that allow for both their protection and management, including lethal control and reduction or elimination from suitable nesting and roosting habitats as a means of limiting their impacts on natural resources (Dorr & Somers, 2012; Dorr et al., 2014; Wires et al., 2005). Thus, the realized ecological niche where *P. auritus* nests do not necessarily represent its potential ecological niche and human disturbance characterizes a portion of the disparity between the fundamental and realized habitat uses of *P. auritus*. Our models include anthropocentric variables that could help increase prediction accuracy for nest site suitability and, therefore, identify regions where management of resident phenotypes might be treated differently. Many of these anthropocentric variables can be altered to some degree through urban planning and natural resource management, points to consider for management goals designed to influence the distribution of *P. auritus*.

Geographic parameters such as prevalence of water bodies and forested land are critical predictors for the distribution of waterbird metapopulations as are anthropogenic parameters such as the quantity and distribution of impervious surfaces (Becker & Weisberg, 2015). These and other parameters can be manipulated through changes in land management practices (Liu & Taylor, 2002). By including model parameters that influence the feeding and nesting success of cormorants, we developed assessments that predict species distribution better than climatic variables alone (Cabral & Kreft, 2012) and inform managers of variables that could be considered for habitat manipulation. For example, connecting and converting undeveloped lands to forested habitat near current and potential nesting sites might reduce the attractiveness of a site for *P. auritus* colonies. Additionally, the removal or alteration of roosting habitat (standing dead cypress trees) in areas where *P. auritus* are undesirable could prevent colony establishment and persistence. Furthermore, parameters such as fish stocking could be explored in greater detail to clarify whether the timing, stocking numbers, richness of species stocked, or size of stocked fishes could be altered to deter or attract *P. auritus*. We encourage managers to consider using SDMs to identify factors that could be manipulated to alter the attractiveness of managed lands to cormorant colonies while still preserving ecosystem services. Models like these could be used by conservationists interested in differentiating between migratory and resident groups in the absence of reliable molecular evidence and where colony establishment is accepted or even desirable.

Here, we demonstrate how readily available environmental variables can be used to develop SDMs that describe the distribution of colonial waterbirds, using double‐crested cormorant nesting habitat as a case study. The model trained on data from Florida successfully identified contemporary nesting sites of *P. auritus* in South Carolina. This information could be used to refine management plans for both migratory and resident cormorant phenotypes in states where the two overlap in geographic distribution. Resource managers can deploy similar methods to identify the current and future distributions of wildlife, particularly where conservation and management of metapopulations differ.

## Conflict of interest

None declared.
