# Supplementary material for: Using species distribution models to define nesting habitat of the eastern metapopulation of double‐crested cormorants
Source: Ecol Evol. 2016 Dec 20;7(1):409–18. doi: 10.1002/ece3.2620 (PMC5215296; doi:10.1002/ece3.2620)
Supplement: Supplementary file 1 [file ECE3-7-409-s001.docx]

## Journal of Ecology and Evolution

## SUPPLEMENTAL MATERIAL

Using Species Distribution Models to Define Nesting Habitat of the Eastern Metapopulation of Double-crested Cormorants

Kate L. Sheehan, Samuel T. Esswein, Brian S. Dorr, Greg K. Yarrow, Ron J. Johnson

Appendix S1 Text and accompanying tables providing information on the sources, methodologies, and interpretations for the spatial layers used to develop multiple species distribution models of Double-crested Cormorant nesting sites.

**Detailed description of model methodology**

### **Nesting Colony Data**

We developed nesting habitat models using data from Minnesota for the migratory phenotype and data from Florida for the resident phenotype. Data for nesting sites in South Carolina were based on colonies documented by SCDNR in 2011 and 2012 (unpublished data, Christy Hand, SCDNR) and by publications reporting contemporary nesting locations (Post and Seals 1991). All count data were converted to presence-only for Maxent model creation and presence/absence for model validation. To convert colony data initially reported as points to polygons that encompassed the entirety of the habitat where each colony occurred, we identified islands, peninsulas, or forested wetlands using a combination of automated and manual techniques in ArcGIS. Nesting sites were determined by dissolving National Hydrography Dataset (NHD water layers into a single object and identifying non-water features (land) smaller than 10,000 km^2^. We overlaid this layer on satellite imagery to confirm island locations, and created island polygons for nesting sites not captured based on water locations, as was the case when rookeries occupied forested wetlands or islands smaller than the spatial resolution of the source dataset (30m x 30m, Figure 1). Nesting count data were then joined with this ‘islands’ layer in order to estimate colony densities (number of birds/km2). Polygons were developed for all sites censused for cormorants (whether or not nests were found), then converted to points at a resolution of 30 m, allowing us to have many points for presence and absence data. Colony polygons were converted to points, corresponding with cell centers, to ensure that distinct habitat differences within each nesting colony could be captured within the Maxent models. For each state, this layer of censused points was named AllNestSites and contained binary information describing presence (1) or absence of (0) of nesting cormorants and nest density for the site. The subset of points where cormorant nests were observed was the layer input to Maxent for analysis, these layers were named NestPresentPoints.

### **Derivation of Parameters**

Because the parameters used for input into Maxent were in a variety of formats (point, polygon, raster, polyline) all layers were converted, when necessary, to raster and snapped to a common registration point with a cell size of 30x30m.

Cells that are located at the boundary of each state could be influenced by habitats beyond the political boundaries of the states assessed here. To overcome this issue, we included a 10 km buffer (merged with the data for the focal state) of all bordering states to develop each data layer. ‘No Data’ pixels were converted to a number which allowed for focal statistical analysis using the ArcGIS for Desktop software. In some cases, such as water area within a 10km radius (WaterA10kSum), the likelihood of any given location to be impacted positively or negatively by the values of other nearby cells was either summed, averaged, or maximized (i.e. focal statistics) at a radius of either 3.5km or 10km. These focal radii represent foraging ranges of cormorants, and by including the three different types of statistics, we could ask different questions. For example, the layer ‘WaterY3.5Max’ is the output of focal statistics on a binary layer (y or n) of waterbodies, where the new cell value is equal to the highest value of all surrounding cells within a 3.5 km radius, effectively indicating whether or not there is a waterbody within 3.5 km of that point. Alternatively, WaterY3.5Sum provides the number of cells within a 3.5 km radius where there was water. In comparison, WaterY10kSum indicates the number of cells within a 10 km radium where there was water. Layers developed with focal statistics were clipped to the shape of the corresponding state so that raster values outside the political boundaries of Minnesota, Florida, or South Carolina became ‘NoData’. This ensured that background data would be selected only for localities where all variables contained data.

### **Using Tables S3**

To repeat our analysis, it is important that layers be appropriately formatted and consistently derived. We provide unique names (column 3-S2, column 1-S3) for each layer created. Three versions of each layer were created: one for each state used in our assessments. Ultimately, this resulted in 40 layers per state, or a total of 120 layers derived in total. Most layers required extensive processing prior to analysis. In some cases, a single transformation or join prepared an object class for conversion to raster and subsequent raster analyses, but in other cases multiple operations were performed before raster transformation.

Step1: For Nesting census data, we created each polygon included in the layer. We constructed a Water layer from multiple layers of waterbody types (river polylines, lake and pond polygons and points), merging them into a single object class. Wetland layers were downloaded for many individual states and they were merged into one wetland polygon file. Because wetland classification types varied among states, we did not retain wetland type in the resulting polygon layer and converted all values to ‘1’. State fish stocking data were downloaded as two different file types, a polygon layer with waterbodies of the state, and a tabular layer with fish stocking data. We performed a spatial join to merge the data in the table to the polygons and inserted a new column to the data table with the calculated geometry (area) of each polygon (see Fig S1). Fish advisory data were downloaded as waterbody polygons and converted to raster using the maximum concentration of the contaminant (or highest warning level) as the raster value. We added 2 columns to the human county population census (polygon) attribute table: one with the calculated county geometric area, and the other with the population density where the population number was divided by the county area (PopDensity). Land use raster data^[[1]](#footnote-2)^ were reclassified to assign all parcels into one of three categories: 2=Forested (codes 41, 42, 43, 91), 3=Undeveloped (codes 31, 81, 71), or 1=Anthropogenic (21, 22, 23, 32, 33, 85). All other land use types were classified as 0.

Step 2-3 (if needed): We joined all polygons in the AllNestSites to a single layer and used the calculate geometry function to estimate the geographic area of each colony site. Once the geometric area of each colony was measured, the density of birds/km^2^ was calculated. Polygons were then converted to raster at a resolution of 30 x 30 m and then converted to point data located at the centroid of each raster cell (‘AllNestSites’). Any points without cormorant observations were removed from a subsequent layer called ‘NestPresentPoints’. These were input into Maxent as presence data. The waterbody object class was converted to raster, resulting in 2 layers, where raster values in one layer represented the geometric area of each waterbody, and raster values of wetland cells in the other layer remained at 1. These WaterA (area) and WaterY (binary) layers were converted to raster where all cells that were not included initially in polygons were coded with a value of 0.

Avian disease data (both density dependent [Cholera and Boltulism] and non-density dependent [Lead poisoning and Pesticide poisoning]) were initially in polygon form; these layers, along with all aforementioned non-point data were converted to raster for additional processing. Polygon datasets that did not require additional manipulation were converted to raster (presence of agricultural lands [Agricultural], and land use change [LandUseChange]– an index where the type of land use either benefitted, was neutral to, or could have compromised the nesting success of DCCO). The intensity of light at night (NightLights), concentration of impervious surfaces (Impervious), and the designation of Native American lands (NativeAmerican) were already in raster form and required no alteration.

Once all the aforementioned datasets were converted to raster, most were reclassified: The WaterA dataset was reclassified using a geometric scale from 0-10, so the smallest value in the original dataset (cells without water) took a value of 0, the largest value became a 10, and all other values converted within this range using geometric scaling. The same 0-10 geometric scaling was applied to the FishStocking, Anthropogenic, LandUseChange, County Population, and PopDensity layers during reclassification. Land use data were split into three layers: Anthropogenic, Forested lands, and Undeveloped lands were classified with a value of 1 in each respective layer and all other land use types were classified as 0. Native American and Agricultural lands were reclassified as either present (1) or absent (0). The density of night lights and impervious surfaces were reclassified using geometric scaling with values ranging from 0 to 5, where the highest concentrations of light at night or impervious surfaces were designated a 5 and the absence of night light or impervious surfaces were designated as a 0.

Once all layers were reclassified, most were converted with focal statistics to adjust the value of each cell to represent the family of cells within either a 3.5 km or a 10 km radius. The avian diseases, wetlands, and land use layers were processed at a 3.5 km radius. Each water layer and fish stocking layer was run at both a 3.5 km and a 10 km radius. The type of focal statistic could be a sum (to give the total area covered by water within the focal radius and the cumulative area of the waterbody that contributes to the focal radius) or maximum value (to represent the presence or size of the largest waterbody) within the focal area. The wetland layer was split into three types of focal statistic at the 3.5 km radius: sum to estimate the area of wetland, average to estimate the prevalence of wetland, and max to estimate whether wetland was present or not. Maximum values for fish advisories and human population variables were estimated within a focal area of 3.5 km.

Calculated values derived from focal statistics were used for model input. Because there were some cells in the climate data with ‘No Data’ we did not perform focal statistics and excluded any points without value from the available cells chosen at random as absence points by the Maxent program.

### Species Distribution Models

We assessed nesting sites of *P. auritus* for influence of environmental parameters (derived parameters) using the Dismo package in R to communicate with the Maxent program. For Minnesota and Florida, we stacked derived variables and a Maxent algorithm was run on presence-only data on the parameter stack (Hijmans and Elith 2013) using the default settings. We used variable contribution outputs to determine the most influential parameters on nesting location predictions. We built models through a series of iterations (5 models for each step), removing environmental variables in the following order: variables that contributed no explanatory power to the model (providing 0% contribution); variables that provided 0.5% or less explanatory contribution; variables that co-varied significantly with highly explanatory parameters.

Figure S1 Flowchart of processing steps used to create fish stocking raster layers input into the Maxent program.

Data Table

Spatial Join

Calculate Geometry

Figure S2 Model development schematic. All derived layers were initially included in state model training. Five replicate models (where different inferred-absence points were used) were run at each step. After the first step, all non-contributing variables (0% contribution to model performance) were removed. After the second step, all poorly-contributing variables were removed (0.5 or less contribution). After the third step, all redundant (variables created from the same initial data or that tested the same type of impact in a different way) variables were removed.


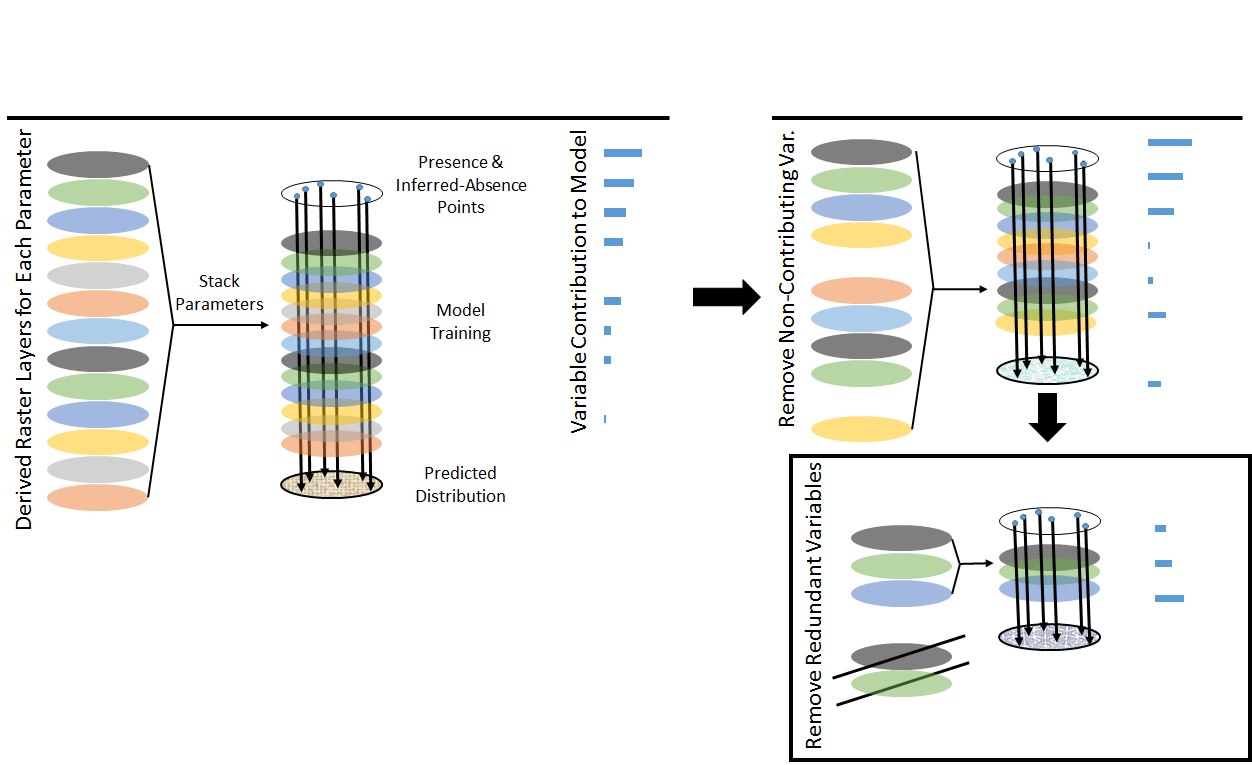


Table S1 Sources of data that used in this study that are available to the public for download

| Data Source | Data Types Downloaded | Website Access |
| --- | --- | --- |
| National Atlas | Human Demography Data | <http://nationalatlas.gov> |
| National Land Cover Database | Land Use Data | <http://www.mrlc.gov>/ |
| National Wetlands Inventory | Wetland Feature Data | <http://www.fws.gov/wetlands/> |
| National Hydrography Dataset | Water Feature Data | <http://nhd.usgs.gov/> |
| United States Environmental Protection Agency | Fish Consumption Advisories | <http://water.epa.gov/> |
| Prism Climate Group | Climate Variable Data | <http://www.prism.oregonstate.edu/> |

**Table S2** Variable description and sources of data used in Maxent models.

| Variable Type | Data Description | Data Source | Data Type | Layer Name |
| --- | --- | --- | --- | --- |
| Water | Presence/Absence | NHD | Raster | WaterY3.5kSUM |
| Water | Presence/Absence | NHD | Raster | WaterY3.5kMAX |
| Water | Presence/Absence | NHD | Raster | WaterY10kSUM |
| Water | Presence/Absence | NHD | Raster | WaterY10kMAX |
| Water | Area(sqkm) | NHD | Raster | WaterA3.5kSUM |
| Water | Area(sqkm) | NHD | Raster | WaterA3.5.MAX |
| Water | Area(sqkm) | NHD | Raster | WaterA10kSUM |
| Water | Area(sqkm) | NHD | Raster | WaterA10kMAX |
| Wetland | Area(sqkm) | USFWS | Raster | WetlandSUM |
| Wetland | Area(sqkm) | USFWS | Raster | WetlandAVG |
| Wetland | Area(sqkm) | USFWS | Raster | WetlandMax |
| NLCD | Presence/Absence | USGS | Raster | Anthropogenic |
| NLCD | Presence/Absence | USGS | Raster | Forest |
| NLCD | Presence/Absence | USGS | Raster | Undeveloped |
| NiteLights | Intensity | USGS | Raster | NightLights |
| Impervious | % imperviousness | USGS | Raster | Impervious |
| Indian Lands | Presence/Absence | National Atlas | Polygon | NativeAmerican |
| Agricultural Lands | % land in agriculture | USDA | Polygon | Ag Lands |
| Land Change | DCCO type | USGS | Raster | LandUseChange |
| Climate | Hundredths of mm | Prism Climate Group | Raster | Precip June |
| Climate | Hundredths of mm | Prism Climate Group | Raster | Precip March |
| Climate | Hundredths of mm | Prism Climate Group | Raster | Precip September |
| Climate | Degrees Celsius | Prism Climate Group | Raster | Min Temp June |

**Table S2 (cont.)** Variable description and sources of data used in Maxent models.

| Climate | Degrees Celsius | Prism Climate Group | Raster | Min Temp March |
| --- | --- | --- | --- | --- |
| Climate | Degrees Celsius | Prism Climate Group | Raster | Min Temp Sept |
| Climate | Degrees Celsius | Prism Climate Group | Raster | Max Temp June |
| Climate | Degrees Celsius | Prism Climate Group | Raster | Max Temp March |
| Climate | Degrees Celsius | Prism Climate Group | Raster | Max Temp Sept |
| Conspecific | Presence nest points | USDA/FFWCC | Raster | BirdDensity |
| Sample Sites | Nests per colony | USDA/FFWCC | Raster | AllNestSites |
| Nest Presence | All sites sampled | USDA/FFWCC | Raster | NestPresentPoints |
| Conspecific | bird density | USDA/FFWCC | Raster | Allocate |
| Conspecific | bird density | USDA/FFWCC | Raster | Euclidean |
| Conspecific | Presence/absence of nests | USDA/FFWCC | Point | PriorDecade |
| Mortality | Avian botulism deaths | USGS NWHC | Polygon | Botulism |
| Mortality | Avian cholera deaths | USGS NWHC | Polygon | Cholera |
| Mortality | Avian lead poisoning deaths | USGS NWHC | Polygon | Lead |
| Mortality | Avian orthophosphate deaths | USGS NWHC | Polygon | Pesticide |
| Fish Advisories | Current mercury advisories | USEPA | Poly, Line, Pt | Mercury |
| Fish Advisories | Current advisories of other pollutants | USEPA | Poly, Line, Pt | OtherPollutant |
| Fish Advisories | Current PCB advisories | USEPA | Poly, Line, Pt | PCBs |
| Fish Advisories | Advisories no longer in effect | USEPA | Poly, Line, Pt | Rescind |
| Human Population | Number of people per county | U.S. Census Bureau | Polygon | CountyPop |
| Human Population | Number of people per sqkm | U.S. Census Bureau | Polygon | PopDensity |
| Fish Stocking | Pounds of fish stocked | MNDNR, FFWCC | Point | LbsFishStocked3.5k |
| Fish Stocking | Pounds of fish stocked | MNDNR, FFWCC | Point | LbsFishStocked10k |
| Fish Stocking | Number of Fish Stocked | MNDNR, FFWCC | Point | FishStockSqkm3.5k |
| Fish Stocking | Number of Fish Stocked | MNDNR, FFWCC | Point | FishStockSqkm10k |

**Table S3** Description of steps taken to derive each variable for the states of Minnesota, Florida, and South Carolina.

| Layer Name | Data Treatment Steps | | | | | | | | |
| --- | --- | --- | --- | --- | --- | --- | --- | --- | --- |
|  | Step 1 | Step 2 | Populate | Poly>Ras | Reclass | Range | Data Type | Focal Stats | |
| AllNestSites | Create Polygon | Join point data | Area | Y | Y | 0/1 | Presence |  |  |
| NestPresentPoints |  |  |  | N | Y | ND/1 | Presence |  |  |
| **Conspecific** |  |  |  | N | Y |  |  |  |  |
| Botulism |  |  |  | Y | N |  |  | 3.5k | MAX |
| Cholera |  |  |  | Y | N |  |  | 3.5k | MAX |
| **Foraging** |  |  |  | N | N |  |  |  |  |
| WaterY3.5kSUM | Merged Waterbodies | Created Waterbody Col. | 1 | Y | Y | 0/1 |  | 3.5k | SUM |
| WaterY3.5kMAX | Merged Waterbodies | Created Waterbody Col. | 1 | Y | Y | 0/1 |  | 3.5k | MAX |
| WaterY10kSUM | Merged Waterbodies | Created Waterbody Col. | 1 | Y | Y | 0/1 |  | 10k | SUM |
| WaterY10kMAX | Merged Waterbodies | Created Waterbody Col. | 1 | Y | Y | 0/1 |  | 10k | MAX |
| WaterA3.5kSUM | Merged Waterbodies | Calc. polygon area | Area | Y | Y | 0 -10 | Geometric Interval | 3.5k | SUM |
| WaterA3.5.MAX | Merged Waterbodies | Calc. polygon area | Area | Y | Y | 0 - 10 | Geometric Interval | 3.5k | MAX |
| WaterA10kSUM | Merged Waterbodies | Calc. polygon area | Area | Y | Y | 0 - 10 | Geometric Interval | 10k | SUM |
| WaterA10kMAX | Merged Waterbodies | Calc. polygon area | Area | Y | Y | 0 - 10 | Geometric Interval | 10k | MAX |
| WetlandSUM | Merged State Wetlands | Created Wetland Col. | 1 | Y | N |  |  | 3.5k | SUM |
| WetlandAVG | Merged State Wetlands | Created Wetland Col. | 1 | Y | N |  |  | 3.5k | MEAN |
| WetlandMax | Merged State Wetlands | Created Wetland Col. | 1 | Y | N |  |  | 3.5k | MAX |
| LbsFishStocked3.5k | Spatial Join |  |  | Y | Y | 0 - 10 | Geometric Interval | 3.5k | SUM |
| LbsFishStocked10k | Spatial Join |  |  | Y | Y | 0 - 10 | Geometric Interval | 10k | SUM |
| FishStockSqkm3.5k | Spatial Join | Calc. waterbody area | Area | Y | Y | 0 - 10 | Geometric Interval | 3.5k | SUM |
| FishStockSqkm10k | Spatial Join | Calc. waterbody area | Area | Y | Y | 0 - 10 | Geometric Interval | 10k | SUM |

**Table S3 (cont.)** Description of steps taken to derive each variable for the states of Minnesota, Florida, and South Carolina.

| Layer Name | Data Treatment Steps | | | | | | | | |
| --- | --- | --- | --- | --- | --- | --- | --- | --- | --- |
|  | 1. Raster Conversion | Step 2 | Populate | Poly to Ras | Reclass |  |  | Focal Stats | |
| **Nesting** |  |  |  |  |  |  |  |  |  |
| Undeveloped | N |  |  | N | Y | 0/3 | Undeveloped land | 3.5k | SUM |
| Forest | N |  |  | N | Y | 0/2 | Forested | 3.5k | SUM |
| Precip June | N |  |  | N |  |  |  |  |  |
| Precip March | N |  |  | N |  |  |  |  |  |
| Precip September | N |  |  | N |  |  |  |  |  |
| Min Temp June | N |  |  | N |  |  |  |  |  |
| Min Temp March | N |  |  | N |  |  |  |  |  |
| Min Temp Sept | N |  |  | N |  |  |  |  |  |
| Max Temp June | N |  |  | N |  |  |  |  |  |
| Max Temp March | N |  |  | N |  |  |  |  |  |
| Max Temp Sept | N |  |  | N |  |  |  |  |  |
| **Anthropocentric** |  |  |  |  |  |  |  |  |  |
| Anthropogenic | N |  |  | N |  | 0 -10 | Anthropogenic | 3.5k | SUM |
| NightLights | N |  |  | N | Y | 0-5 | Geometric | 3.5k | SUM |
| Impervious | N |  |  | N | Y | 0-5 | Geometric Interval | 3.5k | SUM |
| NativeAmerican | N |  |  | N | Y | 0/1 | Reservation Present | 3.5k | SUM |
| Ag Lands | N |  |  | Y | Y | 0/1 | Present | 3.5k | SUM |
| LandUseChange | N |  |  | Y | Y | 0 -10 | Geometric Interval | 3.5k | SUM |
| Lead | N |  |  | Y | N |  |  | 3.5k | MAX |
| Pesticide | N |  |  | Y | N |  |  | 3.5k | MAX |
| Mercury | Y | Cell stats Max |  | N | N |  |  | 3.5k | MAX |
| OtherPollutant | Y | Cell stats Max |  | N | N |  |  | 3.5k | SUM |
| PCBs | Y | Cell stats Max |  | N | N |  |  | 3.5k | SUM |
| Rescind | Y | Cell stats Max |  | N | N |  |  | 3.5k | SUM |
| CountyPop | N |  |  | Y | Y | 0 -10 | Geometric Interval | 3.5k | MAX |
| PopDensity | N | Calc Area | Pop/area | Y | Y | 0 -10 | Geometric Interval | 3.5k | MAX |

1. http://landcover.usgs.gov/classes.php [↑](#footnote-ref-2)
